# Perception of educational environment among medical students in Thailand

**DOI:** 10.5116/ijme.5a4a.1eda

**Published:** 2018-01-26

**Authors:** Wasana Hongkan, Rajin Arora, Roungtiva Muenpa, Parinya Chamnan

**Affiliations:** 1 Medical Education Center,Chonburi Hospital, Chonburi, Thailand; 2 Collaborative Project to Increase Production of Rural Doctor, Ministry of Public Health, Nonthaburi, Thailand; 3 Medical Education Center, Sanpasitthiprasong Hospital, Ubon Ratchathani, Thailand

**Keywords:** Educational environment, Dundee Ready Education Environment Measure, undergraduate students, clinical years, medical schools, Thailand

## Abstract

**Objectives:**

To examine the perception of educational environment among clinical year students in Thailand using Dundee ready education environment measure (DREEM) and identify factors associated with the DREEM scores.

**Methods:**

A total of 2,467 fourth- to sixth-year students from 34 teaching hospitals nationwide responded to a Thai version of DREEM questionnaire. Data on each student’s sex, year of study, size of teaching hospitals and GPAX were collected. Mean total DREEM scores and subscales were calculated and then compared across groups using t-test and one-way ANOVA.

**Results:**

The overall student perception on educational environment was ‘more positive than negative,’ with the mean total DREEM score of 131.1 (SD=17.4). Similar findings were observed in all subscales. Mean total DREEM scores were lower in medium-size than small- and large-size teaching hospitals 129.9 (SD = 18.1), 131.9 (SD = 17.5) and 131.6 (SD=16.4) respectively (F _(2,2422)_ =3.21, p=0.04). Sex and years of study was associated with certain DREEM subscales.

**Conclusions:**

Clinical year medical students in Thailand were satisfied with their academic learning environment, with varying perceptions across different size of teaching hospitals. Repeat assessment of educational environment of medical schools over time is needed for monitoring changes after specific educational interventions being applied.

## Introduction

Educational environment consists of many factors such as physical environment (e.g., classroom and equipment), teachers, colleagues and other student support systems that can motivate the engagement of the learner. ^1^ It is an important part in the curriculum that has been reported to be associated with student’s satisfaction, academic achievement and effectiveness of learning. ^2^ Assessment of student’s perception on educational environment may help provide medical schools with barriers and opportunities for improvement of learning experiences in medical students. ^3^ Educational environment has become an essential area for medical education program evaluation by many authorities such as the World Federation for Medical Education (WFME). ^4^

Many tools have been developed for assessing student perception of educational environment. Most previous studies reported ‘more positive than negative’ learning environment.It is also suggested that perceived educational environment may be related to a number of factors such as student’s sex, study years and academic performance, and characteristics of medical schools. ^5^ ^-^ ^15^ However, most of the previous studies described student perceived educational environment in a single or small number of medical schools; national data were scarce. ^8^ ^-^ ^15^ National data would allow comparisons of learning environment across different medical schools or teaching facilities, hence benchmarking can be done and used as a tool for improvement. There is a lack of evidence to describe educational environment perceived by clinical year students in medical schools in Thailand. Further, it remains unclear if the perception is related to certain characteristics of medical students and teaching facilities.

The objective of the present study was to examine the perception of educational environment among clinical year medical students trained at 34 teaching hospitals across Thailand and to identify factors associated with overall educational environment scores and subscales.

## Methods

### Study design and participants

This was a cross-sectional survey carried out by the Office of the Collaborative Project to Increase Production of Rural Doctors or CPIRD, with a primary aim to study the student perceived educational environment of teaching hospitals under the CPIRD project. Between July 2015 and April 2016, 2,878 fourth- to sixth-year medical students trained in different clinical clerkships in 34 teaching hospitals nationwide were asked to complete a questionnaire. A total of 2,467 students responded to the questionnaire (a response rate of 85.7%). No written informed consent was given by the participants as the survey was considered part of the quality improvement of these teaching hospitals. Before completing the questionnaire, the students were informed about the study objectives and procedures and reassured that research data would be treated anonymously. Ethical approval for the study was obtained from the Ethical Review Board of Chonburi Hospital.

Table 1 shows characteristics of the medical students participating in this survey. Among all participants, 1,424 (58%) were female, and similar numbers of fourth-, fifth- and sixth-year medical students participated. The mean (standard deviation, SD) GPAX of medical students was 3.15 (0.38) out of 4.00, and almost half of the students had a GPAX of 3.01-3.50. A similar number of students were trained in large-, medium- and small-sized hospitals/ medical education centers. Similar proportions of the students were from each of four geographical regions of Thailand.

### Data collection methods

Fourth- to sixth-year medical students were asked to complete a questionnaire, which included two parts: (i) educational environment using a Thai version of the DREEM questionnaire, and (ii) student’s characteristics including sex, year of study, grade point average (GPAX) and hospitals in which they were trained. The DREEM questionnaire is comprised of 50 five-point Likert scale questions on learning environment and atmosphere (0= strongly disagree, 1= disagree, 2= neither agree or disagree, 3= agree and 4= strongly agree), with a total score ranging from 0 to 200. Nine of the 50 items are negative statements. The questionnaire is divided into five subscales: (1) students’ perceptions of learning (SPL), (2) students’ perceptions of teachers (SPT), (3) students’ academic self-perception (SAP), (4) students’ perceptions of atmosphere (SPA), and (5) students’ social self-perception (SSP). Maximum scores for the above DREEM subscales are 48, 44, 32, 48 and 28, respectively. The questionnaire was reported to have high validity and reliability in student nurses (Cronbach’s alpha = 0.91). ^8^ The Dundee Ready Education Environment Measure (DREEM), which was first developed in 1997, is among the most widely used tools. It has been translated and used in many countries mainly in preclinical year students. According to a practical guide by McAleer and Roff, the total DREEM score was interpreted to different conditions of educational environment as follows: 0-50 = very poor, 51-100 = plenty of problems, 101-150 = more positive than negative, 151-200 = excellent. ^16^ A mean score of ≥3.5 for each item was considered real positive, a mean score of 2.0-3.0 indicates that the item could be enhanced and a mean score of ≤2.0 was considered problematic areas.

**Table 1:** Characteristics of medical students participating in the survey (N=2,467)

Characteristic	N (%)
Year of study	
Fourth-year	885 (36)
Fifth-year	819 (33)
Sixth-year	763 (31)
Female (gender)	1,424 (58)
GPAX	
≤2.00	13 (0.6)
2.01-2.50	117 (5.8)
2.51-3.00	571 (28.4)
3.01-3.50	956 (47.5)
>3.50	357 (17.7)
Size of teaching hospitals ^*^	
Small size	713 (29.0)
Medium size	848 (34.4)
Large size	902 (36.6)
Geographic region	
Northern	605 (24.9)
Southern	611 (25.1)
North-eastern	703 (29.0)
Central and Eastern	506 (21.0)

*Teaching hospitals were divided into 3 groups by their size: 1. Large sized hospital teaching 120-180 medical students; 2. Medium sized hospital teaching 90-119 medical students; 3. Small sized hospital teaching fewer than 90 medical students

### Statistical analysis

Characteristics of students and teaching hospitals (medical education centers) were summarized as number (percentage) and mean (SD) for categorical and continuous variables respectively. Average total DREEM scores and subscales were described. The average score for each DREEM item was also examined and ranked; therefore, areas for improvement could be identified. Independent t-test was used to compare the average total DREEM scores and subscales between male and female students, and one-way ANOVA was used to compare the scores across different years of study, sizes of medical, educational centers and geographic regions. The proportions of students perceiving their learning environment ‘excellent’ were compared across different size of medical education centers using chi-square test. A p-value of <0.05 was considered statistically significant.

## Results

### DREEM scores and subscales

The mean total DREEM score was 131.1 (SD=17.3) out of 200. The mean total scores in 34 teaching hospitals ranged from 119.7 (SD=20.4) to 139.7 (SD=14.0). Two thousand and seventy-two students (84%) rated a total score of 101-150, while 253 students (12%) rated a total score of 151-200 and 102 students (4%) reported a total score of below 101. The mean scores (SD) for SPL, SPT, SAP, SPA, SSP subscales were 31.4 (4.2), 30.7 (4.8), 21.4 (3.8), 29.8 (4.8) and 17.7 (3.6), respectively.

Considering each of 50 items of DREEM, eleven items averaged below 2.5, and nine items averaged more than 3.0 ( Table 2 ). None averaged lower than 2.0. Students perceived the most positive on ‘The teachers are knowledgeable,’ ‘Long-term learning is emphasized over short-term learning’ and ‘I have good friends on the course.’ They agreed the least with the items ‘I find the experience disappointing’ and ‘The enjoyment outweighs the stress of the course’.

**Table 2:** DREEM items with an average score of greater than 3.0 or lower than 2.5

Item No	Items	Subscale	Mean score	SD	Median score	Min-Max score
2	The course organizers are knowledgeable	SPT	3.38	0.61	3	0-4
47	Long term learning is emphasized over short-term learning	SPL	3.32	0.67	3	0-4
15	I have good friends in this course	SSP	3.15	0.74	3	0-4
19	My social life is good	SSP	3.13	0.67	3	0-4
30	There are opportunities for me to develop interpersonal skills	SPA	3.11	0.63	3	0-4
31	I have learned a lot about empathy in my profession	SAP	3.02	0.71	3	0-4
18	The course organizers appear to have effective communication skills with patients	SPT	3.02	0.69	3	0-4
7	The teaching is often stimulating	SPL	3.01	0.69	3	0-4
40	The course organizers are well prepared for their teaching sessions	SPT	3.01	0.68	3	0-4
41	My problem-solving skills are being well developed here	SAP	2.48	0.72	2	0-4
36	I am able to concentrate well	SPA	2.47	0.78	3	0-4
12	This course is well timetabled	SPA	2.39	0.87	2	0-4
3	There is a good support system for registrars who get stressed	SSP	2.37	0.92	2	0-4
11	The atmosphere is relaxed during consultation teaching	SPA	2.33	0.92	2	0-4
48	The teaching is too teacher centered	SPT	2.28	0.91	2	0-4
4	I am too tired to enjoy the course	SSP	2.27	1.00	2	0-4
27	I am able to memorize all I need	SAP	2.27	0.83	2	0-4
14	I am rarely bored on this course	SSP	2.27	0.86	2	0-4
35	I find the experience disappointing	SPA	2.13	1.00	2	0-4
42	The enjoyment outweighs the stress of the course	SPA	2.06	0.90	2	0-4

### Factors associated with DREEM scores and subscales

There was no difference in overall perceived educational environment, as measured by total DREEM scores, across years of study (Table 3). However, students in different years of study appeared to perceive their learning environment differently regarding teachers (F_(2,2455)_= 13.14, p <0.001), academic self-perception (F_(2,2453)_= 8.6, p < 0.001), atmosphere (F_(2,2452)_= 7.44, p <0.001), and social self-perception (F_(2,2453)_= 3.91, p <0.02). While sixth- and fifth-year students appeared to perceive more positively about learning environment concerning academic self-perception, social self-perception, and atmosphere, they felt less positive about their teachers than fourth-year students.

Albeit no gender difference in overall perceived learning environment, female students seemed to perceive more positively about their teachers than male students (t = -2.54, p=0.011). However, male students had more positive academic self-perception than their female counterparts (t = 3.53, p<0.001).

Students in the large- and small-sized teaching hospitals/ medical education centers perceived more positively about their educational environment than those in medium-sized hospitals (F _(2,2422)_ = 3.21, p=0.04). Similar findings were observed for teacher (F _(2,2455)_ = 12.93, p<0.001), atmosphere (F _(2,2452)_ = 5.35, p=0.004) and social self-perception subscales (F _(2,2453)_ = 3.53, p=0.029).

The proportion of students perceiving their learning environment ‘excellent’ was highest in small-sized hospitals/ medical education centers (12.1%, 9.9% and 9.7% for small-, medium- and large-sized hospitals respectively, (χ ^2^ (6, N=2425) = 16.08, p=0.010). There was no difference in perceived learning environment across geographical region.

## Discussion

This paper describes medical student’s perception of educational environment in 34 teaching hospitals nationwide using a Thai version of DREEM questionnaire. Overall, the medical students perceived more positively than negatively about their learning environment, although a small number of students felt dissatisfied. Students trained in teaching hospital of different sizes perceived differently about their learning environment, while student sex and year of study were associated with certain subscales of educational environment.

Similar to our findings, previous studies from Australia, Sweden and the United Kingdom reported that there were ‘more positive than negative’ student perceptions regarding learning environment and atmosphere as a whole, with a small proportion of students reporting ‘excellent’ or ‘poor’ learning environment. ^9^ ^,^ ^17^ ^-^ ^19^ A few national-level studies have been done in medical students, and they similarly reported a wide range of global score of learning environment. ^20^ ^,^ ^21^ This observation may be expected due to a great variety of medical schools across the countries. Noteworthy, our study showed a higher average total DREEM score and narrower standard deviations than that from national surveys in Korea and Brazil (131.1 (SD 17.3), 114.0 (SD 21.6) and 119.4 (SD 27.1) respectively).

**Table 3:** Educational environment, measured by total DREEM scores and subscales, reported by clinical year medical students in MOPH hospitals by year of study, gender and size of teaching hospital / medical education centers (MECs)

subscales	Year of study				Gender			Size of teaching hospitals / MECs			
	Year 4 N=882	Year 5 N=816	Year 6 N=757	F _(df1,df2),_ p-value ^*^	Male N=1042	Female N=1416	t _(df),_ p-value ^*^	Small N=710	Medium N=846	Large N=902	F _(df1,df2),_ p-value ^*^
	Mean(SD)	Mean(SD)	Mean(SD)		Mean(SD)	Mean(SD)		Mean(SD)	Mean(SD)	Mean(SD)	
SPL	31.3(4.5)	31.3(4)	31.5(3.9)	F _(__2,2455)_ =0.76, 0.469	31.3(4.5)	31.5(3.9)	t _(__2456)_ =-0.97, 0.329	31.5(4.2)	31.1(4.3)	31.5(4.0)	F _(__2,2455)_ =0.19, 0.015
SPT	31.0(5.0)	31.0(4.7)	30.0(4.7)	F _(__2,2455) =_ 13.14, <0.001	30.4(5.1)	30.9(4.6)	t _(__2456)_ =-2.54, 0.011	31.3(5.0)	30.1(4.9)	30.7(4.6)	F _(__2,2455)_ =12.9, <0.001
SAP	21.0(4.0)	21.4(3.7)	21.9(3.7)	F _(__2,2453)_ =8.60, <0.001	21.7(4.0)	21.2(3.6)	t _(__2454)_ =3.53, <0.001	21.5(3.9)	21.2(3.9)	21.5(3.6)	F _(__2,2453)_ =1.50, 0.224
SPA	29.3(4.8)	30.0(5.0)	30.0(4.0)	F _(__2,2452)_ =7.44, <0.001	29.7(5.0)	29.9(4.6)	t _(__2453)_ =-0.74, 0.457	30.0(4.8)	29.3(4.9)	30(4.6)	F _(__2,2452)_ =5.35, 0.004
SSP	17.5(3.6)	17.7(3.6)	18.0(3.3)	F _(__2,2453)_ = 3.91, 0.020	17.7(3.7)	17.8(3.5)	t _(__2454)_ =-1.01, 0.312	17.4(3.7)	17.9(3.6)	17.8(3.4)	F _(__2,2453)_ =3.53, 0.029
Total score	130.3(18)	131.4(17.2)	131.5(16.6)	F _(__2,2422)_ = 1.23, 0.292	130.8(18.6)	131.3(16.3)	t _(__2423)_ =-0.73, 0.464	131.9(17.5)	129.8(18)	131.6(16)	F _(__2,2422)_ =3.21, 0.040

*p-values for comparison across two and three groups using t-test and ANOVA respectively SPL: students’ perceptions of learning; SPT: students’ perceptions of teachers; SAP: students’ academic self-perception; SPA: students’ perceptions of atmosphere; SSP: students’ social self-perception

Although this does not imply absolute advantages of Thailand’s medical schools over those in the two countries, it suggests that overall learning environment in teaching hospitals in Thailand relatively meet most students’ expectations. It also suggests that educational environment and atmosphere were fairly consistent across 34 teaching hospitals of Thailand.

Student’s perceptions on learning environment and atmosphere provide valuable information for curriculum development and improvement of medical schools. The DREEM questionnaire and similar other tools were designed to describe several aspects of learning environment ranging from teachers, atmosphere, supporting facilities to student’s academic self-perception. Therefore, they can be used to identify strengths and limitations of medical schools, which may form key parts of the framework for improvement of medical schools themselves and to benchmark with other medical schools. As shown in our study, teachers appeared to be one of the key strengths of Thailand’s CPIRD teaching hospitals. This might be because CPIRD particularly have continuously improved its teacher-to-student ratio and faculty development. On the other hand, it seemed that atmosphere and social self-perceptions were among the crucial areas for improvement. To address these issues, a number of interventions may be implemented, such as stress management program, and social and academic support for medical students to develop self-resilience. In addition, student support and guidance such as effective mental health program and advising/ mentoring program may also be beneficial. ^22^ ^,^ ^23^

Student perceptions on educational environment and atmosphere may change over years of study as the result of interaction between student experience and varying challenges over time. In the present study, although overall perceptions on learning environment were consistent across years of study, students’ academic and social self-perceptions and perception of atmosphere were increasingly positive when they advanced to a more senior year. This is in contrast with many previous studies which showed the decreasing trend of DREEM scores over years of study. ^9^ ^,^ ^10^ ^,^ ^24^ ^,^ ^25^ This may suggest different student’s ability to adapt themselves to evolving challenges such as clinical tasks and assignments when advancing through more senior years, or may simply reflect discrepancy in medical curriculum in these countries. Another explanation for such findings might lie upon the ability of a tool to capture complete aspects of clinical training in different years of study. Questionnaires to measure educational environment should be more contextualized and specific to clinical years of medical study. One example is a tool called Teaching and Learning Climate or TLC, which includes additional items on ward environment, colleagues such as residents, house officers and nurses, and learning experience related to clinical tasks and assignments. ^26^

Gender inequality in perceptions of educational environment has been consistently reported. Similar to previous several studies, ^9^ ^,^ ^12^ our study showed that female medical students had more positive perception on learning environment than their male counterparts, particularly on teachers. A more positive perception of teachers in female students might be due to different learning styles of the two sexes or simply gender bias towards female students in teaching and evaluation. ^27^ ^,^ ^28^ In addition, more positive academic self-perception in male students may partly be because male medical students seemed to be more confident in their academic performance than female students. ^29^

Size of medical schools may have influence on educational environment, with some evidence suggesting favorable learning environment in small-sized medical schools. Similar to the previous studies in Australia and Canada, ^13^ ^,^ ^17^ ^,^ ^30^ ^-^ ^32^ our study found that students in small-sized medical schools and training facilities perceived more positively about their learning environment than those from a larger medical school. This may be explained by that small sized teaching hospitals may provide closer teacher-student and student-student interaction. In addition, large sized teaching hospitals are usually equipped with highly sophisticated medical technologies and taught by subspecialty medical staffs. This may be less relevant to training of medical general practitioners than small sized hospitals. Hence, students at smaller medical training centers may be more satisfied with their educational experience than those trained at the larger training centers. ^13^ ^,^ ^17^

CPIRD is a good demonstration that using existing health services as training facilities helps enhance student’s experience in community-oriented competencies and interprofessional skill. ^33^ Previous studies suggests that medical teaching in service hospitals may also help equip students with better clinical competency as compared to teaching in large university hospitals. ^33^ ^-^ ^35^ Training in real healthcare services may provide students with hands-on experience on clinical practice and procedures, which are more relevant to their future career practice. Many authorities have suggested that doctors should be produced in real health service system with enhancing community-oriented competencies such as teamwork skills and being a change agent. ^36^ ^,^ ^37^

Our study has some limitations. First, as the DREEM questionnaire is a tool to assess the perception of undergrad students concerning general learning environment, it might not reflect the whole and true picture of clinical teaching environment and climate. A more clinical-specific tool may be needed. Additionally, as this survey was carried out at a certain period of the year when students were on different clerkship rotations, the results might have been different if the study had been done at the different time of academic year. Furthermore, although the DREEM questionnaire has been validated in Thai student nurses, using this tool in students in different majors and faculties may have altered the results. As this survey was conducted solely in the teaching hospitals under a special CPIRD project, it may not represent the educational environment of all medical schools in Thailand.

## Conclusions

Perception of learning environment in clinical year students under Thailand’s CPIRD project was more positive than negative, with differences in many aspects of learning environment between sexes, years of study and size of teaching hospitals. Key areas for improvement of Thailand’s medical schools included stress management, social and academic support systems. Repeat assessment of educational environment of medical schools or departments over time is needed for monitoring changes after specific educational interventions being applied. A tool more specific to clinical year teaching may be needed.

### Acknowledgements

We gratefully acknowledge contributions of 34 Medical Education Centers under the CPIRD project.

### Conflict of Interest

The authors declare that they have no conflict of interest.
